# Machine Learning for Anxiety Detection Using Biosignals: A Review

**DOI:** 10.3390/diagnostics12081794

**Published:** 2022-07-25

**Authors:** Lou Ancillon, Mohamed Elgendi, Carlo Menon

**Affiliations:** 1 Biomedical and Mobile Health Technology Lab, ETH Zurich, 8008 Zurich, Switzerland; lancillon@student.ethz.ch (L.A.); carlo.menon@hest.ethz.ch (C.M.); 2 Department of Computer Science, ETH Zurich, 8092 Zurich, Switzerland

**Keywords:** digital health, anxiety biomarkers, physiological measures, wearable devices, digital psychological assessment

## Abstract

Anxiety disorder (AD) is a major mental health illness. However, due to the many symptoms and confounding factors associated with AD, it is difficult to diagnose, and patients remain untreated for a long time. Therefore, researchers have become increasingly interested in non-invasive biosignals, such as electroencephalography (EEG), electrocardiogram (ECG), electrodermal response (EDA), and respiration (RSP). Applying machine learning to these signals enables clinicians to recognize patterns of anxiety and differentiate a sick patient from a healthy one. Further, models with multiple and diverse biosignals have been developed to improve accuracy and convenience. This paper reviews and summarizes studies published from 2012 to 2022 that applied different machine learning algorithms with various biosignals. In doing so, it offers perspectives on the strengths and weaknesses of current developments to guide future advancements in anxiety detection. Specifically, this literature review reveals promising measurement accuracies ranging from 55% to 98% for studies with sample sizes of 10 to 102 participants. On average, studies using only EEG seemed to obtain the best performance, but the most accurate results were obtained with EDA, RSP, and heart rate. Random forest and support vector machines were found to be widely used machine learning methods, and they lead to good results as long as feature selection has been performed. Neural networks are also extensively used and provide good accuracy, with the benefit that no feature selection is needed. This review also comments on the effective combinations of modalities and the success of different models for detecting anxiety.

## 1. Introduction

Anxiety disorders (ADs) are the most common type of mental illness in the world, affecting 264 million people worldwide [1]. The clinical features of AD include considerable and persistent uneasiness, as well as autonomic nerve activity excitation, and excessive vigilance, all of which are linked to the nervous system [2]. ADs can be categorized as a generalized anxiety disorder (GAD) [3], panic disorder [4], or social anxiety disorder [5].

GAD is characterized by a combination of somatic and mental symptoms, including tremors, muscular tension, sweating, and stomach discomfort, as well as restlessness, sleeplessness, inattention, memory problems, irritability, high sensitivity, and palpitation [3]. Due to the broad range of symptoms, most patients suffering from anxiety have not been diagnosed; therefore, they do not receive adequate treatment [4]. While psychiatrists may now determine whether a patient has an AD based on clinical symptoms, such as self-assessment and pathophysiological reports, these might be erroneous or incorrectly stated.

Traditional psychophysiology studies (i.e., those that do not use machine learning) frequently show no major correlations between physiological parameters and anxiety levels, whereas other research [5] that uses machine learning techniques shows that anxiety recognition through physiological analysis is possible. These challenges and inconsistencies have prompted researchers to create novel technologies to improve well-being while lowering morbidity, mortality, and healthcare expenditures. Different biomarkers, including respiration (RSP), electrocardiogram (ECG), photoplethysmography (PPG), electrodermal response (EDA), and electroencephalography (EEG), can be used to detect physiological responses related to stress and anxiety [6].

The electrical activity of brain neurons in the cerebral cortex or scalp can be recorded using EEG [7]. This approach is ideal for researching the electrophysiological and cognitive states of the human brain because it provides an immediate assessment of the underlying neural activity with a high temporal resolution of a few milliseconds [8].

Mental stress has been measured using ECG signals [9] or PPG, which is an optical measurement of arterial volume using a single photodiode. ECG and PPG signals may be used to extract heart rate (HR) and heart rate variability (HRV). However, according to Jan et al. [10], PPG is a more convenient way to measure HRV than ECG at rest since respiration could be a confounding factor in HRV evaluation. HRV analysis is generally performed using ECG recordings that are longer than 24 h (long-term HRV) or less than five minutes (short-term HRV). Because the general physiological regulations of the individual are represented over this period, the 24 h records are regarded as having a high degree of accuracy. Nevertheless, short-term HRV analysis is thought to be more practical because of its simplicity of use and reduced analysis of time, although it is highly dependent on the window length of the processed ECG signal [11,12,13,14]. Two of the studies [15,16] that are part of this review used ultra-short HRV as a feature (<5 min) to detect anxiety. The key benefit of ultra-short HRV is that it is well suited for mobile applications; consequently, it generates data quickly with a significantly shorter recording time than standard methods [12].

RSP is known to be an indication of psychological stress and anxiety [17,18] and can be influenced by emotional events. Breathing rate, which is determined by measuring the number of breathing cycles per minute, rises when tension or worry increases, resulting in hyperventilation in severe cases [19]. Finally, EDA is a measurement of changes in the skin’s electrical conductance as a result of sweat generation. It has two parameters, skin conductance level, and skin conductance response, and it is extensively utilized as a stress and anxiety indicator [20].

Other studies not discussed in this review aimed to detect anxiety from audio signals, written texts, or functional magnetic resonance imaging (fMRI). The fMRI mechanism eliminates the potential disadvantages associated with the task frameworks of other modalities. However, in comparison to these other modalities, fMRI is somewhat expensive, has a low temporal resolution, and its data are very sensitive to head movements [21,22].

These different biomarkers (ECG, EEG, RSP, and EDA) are used as inputs to machine learning algorithms. The same pipeline was applied in almost all the reviewed articles. First, feature selection was performed using probabilistic distributional clustering (PDC), phase lag index (PLI) algorithms, and sequential feature selection, among other related approaches. Then, classification of anxiety into two (no anxiety vs. anxiety) or three classes (low vs. mid vs. severe anxiety) was performed using a convolutional neural network (CNN), long short-term memory (LSTM), one-vs-one (OVO), random forest, or support vector machine. The aim of this review was to provide an overview of the different combinations of features and models for each type of signal, compare them, and determine which one leads to better results.

## 2. Method

We used the PubMed, IEEE, and Embase databases to conduct the literature searches. We opted to include research from the most recent decade—that is, papers published between 1 January 2012 and 31 March 2022—since we were interested in the most current technological breakthroughs, specifically applying artificial intelligence and machine learning to biosignals. We used a mix of medical subject heading phrases in our search, including “anxiety”, “panic disorder”, “phobia disorder”, as well as the general terms “detection”, “prediction”, “machine learning”, “artificial intelligence”, “signals”, “biosignals”, and “biomedical signals”. The advanced feature in PubMed allows for all derivatives of the keyword to be searched; for example, it does not limit itself to “detection” but also searches for “detect” [All Fields] OR “detectabilities” [All Fields] OR “detectability” [All Fields] OR “detectable” [All Fields] OR “detectables” [All Fields] OR “detectably” [All Fields] OR “detected” [All Fields] OR “detectible” [All Fields] OR “detecting” [All Fields] OR “detection” [All Fields] OR “detections” [All Fields] OR “detects” [All Fields].

## 3. Results

**Figure 1:**
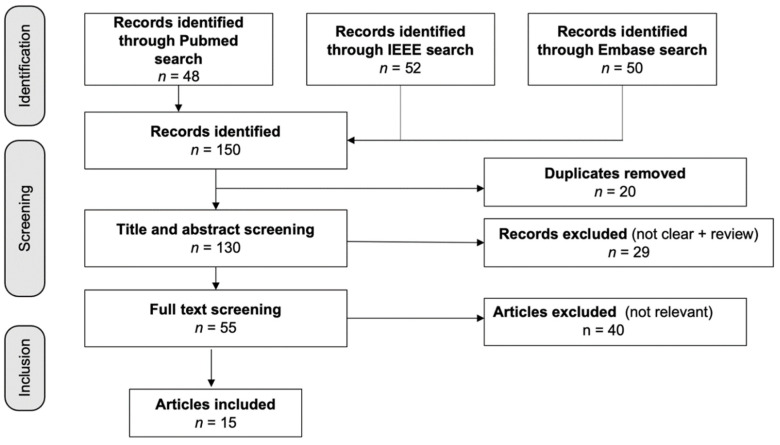
Flow diagram of the included studies. Fifteen studies were identified from 150 articles in the initial database search.

Figure 1 shows the 150 articles that were found in the database search; three duplicates were eliminated. A total of 29 items were rejected after the titles and abstracts were screened. Following the full-text screening, 40 papers were dropped for failing to fulfill the inclusion criteria, which consisted of not mentioning AD, having unclear results, or having an unsuitable research design. For our analysis, we looked at the remaining 15 studies, four of which were based on EEG, two of which were based on EEG plus other biosignals, and nine of which were based on a mix of biosignals, such as ECG, RSP, and EDA.

The literature review returned 15 scientific publications. To compare the articles, we attended to the number of participants; the signal types; the number of classes, experiments, features, and algorithms; and the best accuracy for each.

**Figure 2:**
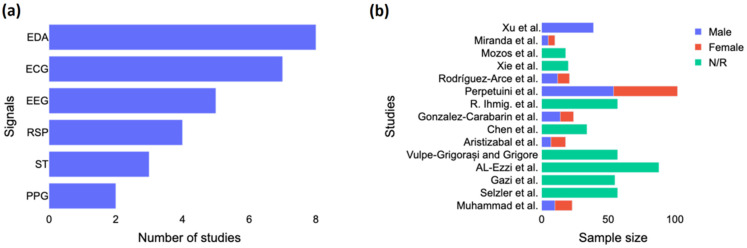
Study characteristics. (**a**) Number of studies investigated each biosignal for anxiety detection and (**b**) Gender breakdown per each study. EDA = electrodermal activity; ECG = electrocardiogram; EEG = electroencephalography; RSP = respiration; ST = skin temperature; PPG = photoplethysmogram; N/R = not reported. The included studies are [15,16,23,24,25,26,27,28,29,30,31,32,33,34,35].

As seen in Figure 2a, EDA was the signal most often used in the studies, followed by ECG and EEG. The studies were usually based only on EEG, and they generally did not combine EEG with other signals, except for Gonzalez-Carabarin et al. [23], who combined EEG with ECG, and Xu et al. [24], who combined EEG with ECG, EMG, and EDA. Figure 2b shows the sample size by study according to the gender of the participants. We noted a majority of male participants in the studies, and more than half did not report the gender of their participants. Only two studies, including those by Al-Ezzi et al. [25] and Perpetuini et al. [26], used a sample size of more than 80 subjects. Four studies had around 55 to 57 subjects, three had between 20 and 40 subjects, and the rest had less than 20 subjects.

**Figure 3:**
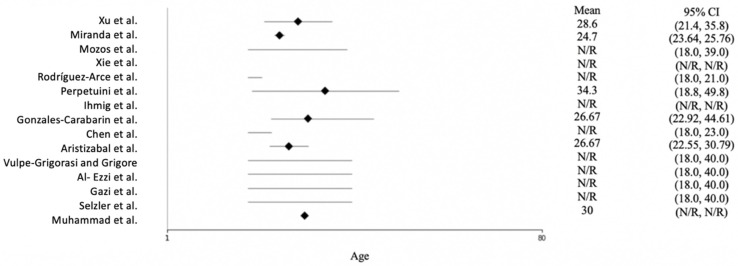
Forest plot of the age of the participants for each study. N/R = not reported. The included studies are [15,16,23,24,25,26,27,28,29,30,31,32,33,34,35].

Figure 3 shows the age distribution of the participants across the studies. Most of the studies reported only the age range of the subjects, not the mean age. Rodríguez-Arce et al. [27] and Chen et al. [28] conducted their studies with students; therefore, they used young participants ranging in age from 18 to 23. The mean age across the studies was approximately 30, and the oldest subject was 49.8 years of age.

## 4. Experiments

We observed two main anxiety classifications. First, most of the publications on EEG focused on a healthy control group and people who suffered from AD. They aim to differentiate between participants with and without AD. Publications about other biosignals only included a health control group or an AD group, and the signals were classified into states of anxiety and rest periods.

Ihmig et al. [15], Gazi et al. [29], and Selzler et al. [30] belong to this second category and focused only on phobic participants. Specific phobia is a common mental disorder that affects about 7.4% of the population at least once in their lives. One of the most prevalent sorts of specialized phobias is the pathological fear of spiders. Patients who present with phobic indications experience severe physical anxiety symptoms, such as tachycardia, sweating, and shortness of breath. In all investigations employing ECG, RSP, and EDA, these signals were recorded during exposure therapy, which is the prevalent approach for the treatment of specific phobias. The patients alternated between phases of rest and phases of exposure, in the latter of which they were confronted with the feared object under controlled conditions.

Most of the other experiments used task and rest period cycles, stress-inducing protocols, or the Trier Social Stress Test (TSST) [36]. All these studies had the same aim of putting the participant in an uncomfortable situation so that he/she would feel anxious.

In order to train machine learning models, it is necessary to assess the ground truth. For this purpose, most of the studies were subject-based and used State-Trait Anxiety Inventory (STAI) labeling. To evaluate the participants’ anxiety levels, they were asked to fill in a questionnaire during or after the experiment. The STAI questionnaire is divided into two sections. The state section measures state anxiety (i.e., how an individual feels right now), and the trait section measures trait anxiety (i.e., how an individual feels generally). Another method used by Gazi et al. [29] and Miranda et al. [31] consisted of labeling the time-series data according to the video clips stimulating anxiety and no-anxiety events.

## 5. Pipeline

**Figure 4:**
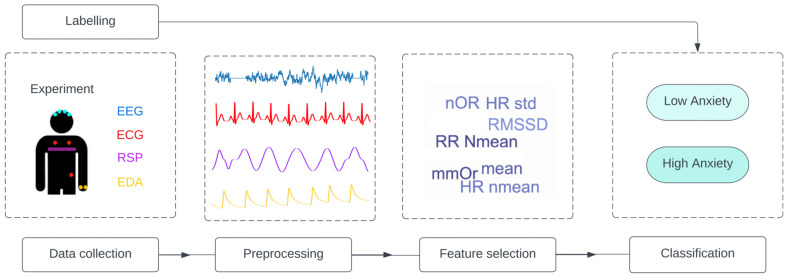
General pipeline applied in all studies. The studies follow the same structure: data collection from one or a combination of signal is performed, and the data are labelled. Then, the data are preprocessed, and feature selection is applied to obtain the input of the classification model. EEG = electroencephalography; ECG = electrocardiogram; RSP = respiration; EDA = electrodermal activity; Nmean = normalized mean; RMSSD = root mean square of successive normal-to-normal interval differences; HR: heart rate; LF/HF = ratio of low frequency to high frequency; NFD = mean of the absolute values of the normalized first differences; NOR = number of orienting responses; mmOR = mean magnitude of orienting responses; PNN50 = proportion of NN50 divided by the total number of NN (R-R) intervals; IBI = inter-beat interval; ACF = autocorrelation function; Ti = inspiration time; Te = expiration time; SD = standard deviation.

Various signals and experiments were used and applied in the reviewed studies, but we observed the same global pipeline in all of them, as shown in Figure 4.

## 6. Features

Feature selection is a key factor in the development of robust classification models. For example, as seen in one study [27], the same algorithm (SVM) had an accuracy of 98% with six features and an accuracy of 86% with 13 features. In contrast, an accuracy of 94% was achieved using RF with 13 features, but the accuracy was only 88% with six features. In this study [27], feature selection was performed using a Student’s t-test.

**Figure 5:**
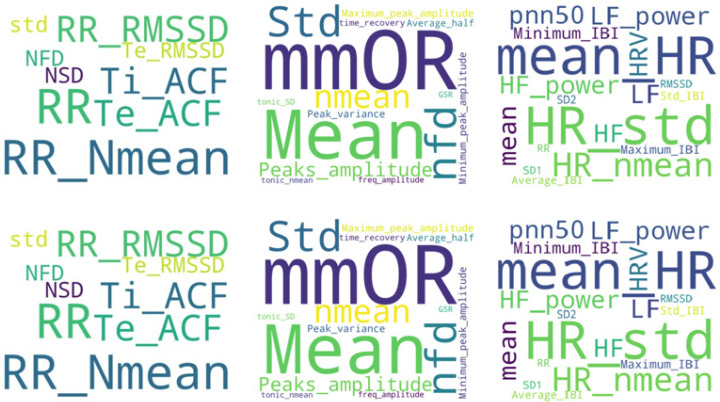
RSP, EDA, and ECG features as the most often used in the reviewed literature. Nmean = normalized mean; RMSSD = root mean square of successive normal-to-normal interval differences; HR = heart rate; LF/HF = ratio of low frequency to high frequency; NFD = mean of the absolute values of the normalized first differences; NOR = number of orienting responses; mmOR = mean magnitude of orienting responses; PNN50 = proportion of NN50 divided by the total number of NN (R-R) intervals; IBI = inter-beat interval; ACF = autocorrelation function; Ti = inspiration time; Te = expiration time; SD = standard deviation.

Sequential feature extraction is another method applied by Ihmig. et al. [15] to select the most significant feature subset. This method starts with an empty set and sequentially adds a candidate feature until a given criterion, the accuracy of each classifier, is fulfilled. Thus, the sequential function stops adding new features when there is no further improvement in accuracy. In another study, Gazi et al. [29] employed a permutation approach to quantify feature importance. Specifically, this consisted of randomly permuting feature *j* ∈ 1, 2, …, *J* of the testing set *K* = 30 different times and averaging the resulting accuracy decreases across all iterations of *k* ∈ 1, 2, …, *K*. This process was then repeated for each of the *J* = 31 features. In addition to this analysis, six experiments (i.e., hyperparameter optimization, training, and testing) were conducted to assess the efficacy of each of the three signal features. The results showed that RSP or ECG signals alone do not allow for any conclusions to be drawn, but when combined with EDA, this approach achieved the best performance. Figure 5 shows the most frequently used features for the most frequently used signals.

Regarding EEG signals, other specific methods for feature selection were used. For instance, Xie et al. [32] applied the PLI connectivity algorithm, which is a commonly used method in undirected networks. This algorithm calculates phase synchronization. The main purpose of PLI is to avoid the influence of common sources and obtain reliable phase synchronization prediction values—that is, to eliminate phase lock. The adjacency matrix obtained by the PLI algorithm was then converted into a binary matrix that determined whether there was an edge choosing an appropriate threshold, which was 0.04 in this experiment. The brain network (BN) was used as an input for the CNN-2, DBN, and LDA algorithms.

Instead of the BN, prefrontal lateralization can be used. This approach is actually one of the most commonly used methods to evaluate patients with anxiety and depression. The score is defined by the equation of *InR*–*InL*, where *R* is the power spectral density of each band in the right brain, and *L* is that of the left brain. If the score is positive, the activity is stronger in the left prefrontal lobe than in the right prefrontal lobe. If the score is negative, there is no left lateralization. As seen in Table 1, BNs perform better with CNN than with prefrontal lateralization, but CNN is better when combined with DBN and LDA.

## 7. Models

Neural networks are widely used, particularly CNNs, which are applied to both EEG and a combination of physiological signals. When working on EEG, a two-dimensional (2D) CNN was used [21,32]; for other signals, a one-dimensional (1D) CNN was used [16,33]. CNN is a multilayer perceptron with several convolution-pooling layers and fully connected layers at the output. Input features are convolved with multiple-dimensional filters in the convolution layer and sub-sampled to produce a smaller scale in the pooling layer. The shared network weights and filters in the convolution layer are learnable through the back-propagation algorithm, which minimizes classification errors.

SVM [27,29,31] and RF [29,30,34] were the predominant algorithms used in the remaining studies. The RF consists of many individual decision trees that work as ensembles. Every tree produces a class prediction, and the class receiving the most votes becomes the model’s prediction. The main difference between bagging and RF is that, in RFs, only a subset of features is selected at random out of the total, and the best split feature from the subset is used to split each node in a tree, unlike in bagging, where all the features are considered for splitting a node. In contrast, SVM is based on the idea of finding a hyperplane that best divides a dataset into two classes.

The two studies combining EEG with other signals first applied K-means [24] and then another cluster-wise classification [23]. On the one hand, Xu et al. [24] used K-means to divide the subjects into different categories, and then regression analysis was conducted using the generalized regression neural network (GRNN) for individual clusters based on the training dataset belonging to the task load and recovery stages. This led to a set of K GRNN models that minimized the cluster-wise error. On the other hand, Gonzalez-Carabarin et al. [23] used unsupervised learning to cluster the EEG features into stress and non-stress periods (plus an extra cluster to categorize data points that were not part of previous categories) and then applied supervised learning. As seen in Table 1, for the same experiment, classification into two categories was more accurate than classification into three categories; thus, it was still difficult to accurately classify levels of anxiety. Finally, in many cases, a validation technique, such as leaving one out or 10-fold cross validation, was applied. Note that the impact of using different cross-validation methods was not discussed.

The best performance for RF was achieved with EEG, then with a combination of ECG and EDA, and, finally, with a combination of ECG, EDA, and RSP. However, this result contradicts the results of Gazi et al. [29], who found an accuracy of 78% with a combination of ECG and EDA and 85% with a combination of ECG, EDA, and RSP. A combination of skin temperature (ST), EDA, RSP, and HR led to the best performance using SVM, followed by a study [28] using only EEG and a study [23] that combined ECG and EEG. For neural networks, the best accuracy was obtained with the study [33] that combined EDA, PPG, and ST. Good performance corresponding to accuracy above 85% was also achieved with EEG or EEG combined with ECG, EDA, and EMG. In contrast, a study [16] using ECG, ST, and RSP had lower accuracy results of 77%.

As mentioned previously (Figure 2), the most used signals were ECG, EDA, and RSP. However, it is also interesting to look at the influence of anxiety on skin temperature. Aristizabal et al. [33] did not find a significant effect of anxiety on the ST. According to them, the body temperature can give information about the intensity of the stress response but is also highly influenced by environmental conditions such as the temperature or the humidity and is, therefore, more suitable for laboratory studies. Rodriguez-Arce et al. [27] has a more nuanced finding since an anxious-induced task significantly influenced two out of four skin temperature features they used.

## 8. Discussion and Future Directions

In this review, we investigated the detection and classification of anxiety using biosignals. The feature selection is one of the critical elements of this classification as it has a direct impact on accuracy. As seen in Figure 4, the researchers followed approximately the same pipeline applying various machine learning algorithms to biosignals extracted during one of the two main experiments discussed previously.

As a positive point in this review, we can note the diversity of approaches used in the mentioned studies. Indeed, several experiments are used but also several machine learning algorithms and biosignals. This gives the readers a wide range of potential directions. However, this strength also has its limits because if there are many possible combinations, only a few are exploited, and the researchers are finally interested in the same variety of experiments with the same model and the same signals, making it difficult to compare.

**Figure 6:**
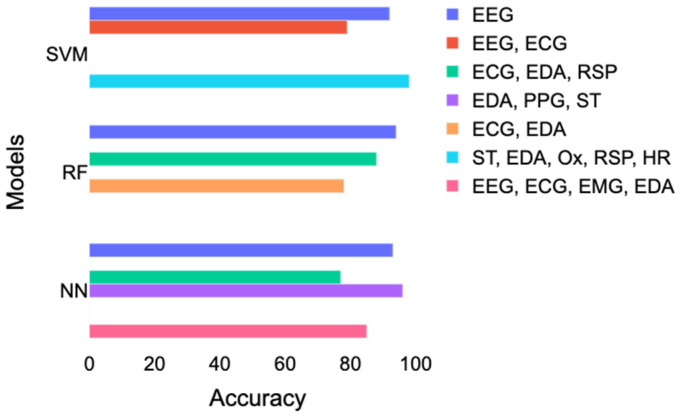
Comparison of the accuracy achieved with SVM, RF, and neural networks according to different combinations of signals. EDA = electrodermal activity; ECG = electrocardiogram; EEG = electroencephalography; RSP = respiration; ST = skin temperature; PPG = photoplethysmogram; Ox = oximetry; HR = heart rate; SVM = support vector machines; RF = random forest; NN = neural networks.

Table 1 and Figure 6 reported different accuracy results using more or less similar combinations of signals. Here is a breakdown of the inconsistencies that led to this issue:

- Small sample sizes;
- Omission of a discussion about confounding factors, such as psychiatric and medical comorbidity;
- Limited information on the subjects’ medication intake status before running the study;
- Lack of information about what kind of AD the patients had;
- Limited information on the genders and ages of the participants;
- Limited information about the feature selection and exact features used;
- Many different combinations of signals and machine learning models were used, which made comparisons difficult;
- Divergence in the classification scheme: general as opposed to person-specific classification;
- Lack of differentiation and comparison between anxiety and anxiety disorder.

When conducting the literature search, it was difficult to find studies on AD instead of simple stress. It was also difficult to find a consistent definition of anxiety in all the studies. It should be noted that up to 60% of AD patients have comorbid depression [34]. As a result, distinguishing between people with and without mental comorbidity is challenging. Moreover, in many studies, there was a lack of information about which type of anxiety was detected. This is troubling because the symptoms are not necessarily the same, and it would be valuable to determine which features are more impacted by which kind of anxiety.

Two studies [23,27] used small sample sizes from a student population characterized as young, active, and healthy. One might therefore question the usefulness of studies such as this performed on a non-representative sample in real life. However, according to Perpetuini et al. [26], no correlation between state anxiety and age was found, but a significant difference between males and females was found for state anxiety.

A distinction must also be made between studies that use general classifications relying on the data of a group of people as a training set but test the model on a different set of people, as well as the person-specific classification, which uses samples from the same participants for the training and testing. All results reported in Table 1 are from general classification studies, except for Chen et al. [28]. Mozos et al. [35] and Gonzales-Carabarin et al. [23], who used both kinds of classifications, came to the same conclusion: general models perform worst in comparison with the subject-oriented model. This observation can be inferred from the fact that subtle features, as used in all the studies of this review, are highly dependent on the individual.

The comparison between anxiety and anxiety disorder is one of the limitations of this review. Indeed, some studies [23,24,26,27,31,35,37], focus on detecting anxiety as an induced experience in danger, whereas others [15,16,19,25,26,28,32] aim to detect ADs subjects which need a timeline to be categorized, from health control. 

Finally, due to limited information about the feature selection and exact features used, it was not possible to compare the efficiency of the different methods since no study (except for that of Xie et al. [32]) used multiple feature selection for the same signals and algorithm. We encountered a similar issue with the models used in the studies. Indeed, having many different combinations of signals across the studies made it difficult to draw conclusions about the advantages of one type over another or to compare the performance of the models.

Wearable technologies that analyze and forecast ECG, EDA, RSP, and EEG waveforms in real time might enable a more thorough examination of AD during the day. As a result, the role of machine learning algorithms that can detect and forecast AD patterns are likely to increase in clinical medicine and outpatient care. The use of biosignals to detect (or monitor) different types of AD is a relatively new area. Indeed, only three of the 15 papers included in this review were published before 2020. Because the topic is challenging, the recent attempts used a small and less-representative sample size. However, we have seen an increase in interest in this topic, and it is thus necessary to propose the following recommendations for researchers:

- Collect biosignals from a large number (>100) of study subjects; a pure control group and a pure AD group (with no confounding factors) need to be used for study validation.
- Have more diversity in the subjects in terms of age and gender.
- Ensure consistency and more detail in diagnosing the participants and have the project monitored by a clinician from start to finish of the pipeline (i.e., from the selection of participants to the interpretation of the results).
- Compare studies on various ads to determine if the type of anxiety has an impact on the results and whether the selected features can detect it.
- Include more information about the feature selection and the features used.

We emphasize that these recommendations be followed, as most studies did not determine or achieve them. Having a larger number of study subjects would allow for more data to be obtained and would ensure that studies provide indisputable evidence. Moreover, the consistency of the participants’ diagnoses and signal processing is part of what it means to have a complete dataset. Furthermore, studies should focus on only one type of anxiety and have doctors select the participants, ensuring that they are in good health or have the ability to assess their own levels of anxiety. Clinicians should also check how the experiments are performed and help interpret the results. Regarding papers that do not use neural networks, it would be interesting to compare the feature selection methods on the same signals. As a future direction, it would be interesting to measure the effect of therapies using biosignals.

## 9. Conclusions

The main markers of human well-being employed in clinical settings are features taken from ECG, EDA, EEG, and RSP signals. Early discovery and intervention in cases of AD are critical since any mental condition may be improved with early recognition and care. In this study, we examined methods for detecting and predicting AD utilizing a combination of biosignals. Most of the experiments were conducted under controlled conditions during different sessions. Despite the fact that the trials were controlled and did not fully reflect real activities, they are valuable scientific attempts in the field since they provide a consistent and safe procedure for creating a stressful atmosphere. This is the natural first step in verifying any new system in a controlled environment such as this. A personal interview is also an example of a social activity that many individuals may encounter.

The review also showed the feature importance and benefits of multi-modality. The results suggest that methods that incorporate a combination of ECG and EDA signals should be encouraged. Models such as SVM and RF are widely used and achieve good performance, but their results depend on the features used, and they require strong feature selection methods. Neural networks or Adaboost also lead to good results without the need for feature selection, which is performed directly during training. Generally, the results of the classification presented here demonstrate that some methods and analyses already provide useful tools for AD prediction. In the long run, this may allow other researchers to consider, for example, the effects of real-time feedback and even identify specific triggers that lead to high and inappropriate levels of anxiety.

## Figures and Tables

**Table 1:** Summary of the findings of the 15 papers included in this review. EEG = electroencephalography; ECG = electrocardiogram; EDA = electrodermal activity; EMG = electromyography; RSP = respiration; ST = skin temperature; PPG = photoplethysmogram; HR = heart rate; HC = healthy control; AD = anxiety disorder; HAM-A = Hamilton Anxiety Rating Scale; SAM = Self-Help for Anxiety Management; SB = subject based; STAI = state-trait anxiety inventory; NE = natural experiment; TSST = Trier Social Stress Test; GRNN = generalized regression neural network; SVM = support vector machine; BN = brain network; PL = prefrontal lateralization; LDA = latent Dirichlet allocation; CNN = convolutional neural network; DBN = deep belief network; OVO = one-vs-one; KNN = K-nearest neighbor; GLM = generalized linear models; RF = random forest; DT = decision tree; N/R = not reported.

Publication	Participant (HC: AD)	Signal Type	Experiment	Categories	Labeling	ML Algo	Validation	Accuracy (%)
Muhammad et al. (2022) [34]	23 (23: 0)	EEG	Exposure therapy	Low/high low/norm al/medium/high	HAM-A + SAM	Random Forest	Leave-one-out cross validation	9492
Selzler et al. (2021) [30]	57 (0: 57)	ECG, EDA	Exposure therapy	Low/high low/medium/high	SB	Random Forest	10-fold cross validation	7860
Gazi et al. (2021) [29]	55 (0: 55)	ECG, EDA, RSP	Exposure therapy	Anxiety/no anxiety	Video levels	Random Forest	Leave-one-out cross validation	88
AL-Ezzi et al. (2021) [25]	88 (22: 66)	EEG	Social performance task	Mild/mode rate/severe	N/R	CNN + LS TM CNN LSTM	N/R	939,186
Vulpe-Grigorași and Grigore (2021) [16]	57 (0: 57)	ECG, ST, RSP	Exposure therapy	Anxiety/no anxiety	N/R	1D-CNN	N/R	77
Aristizabal et al. (2021) [33]	18 (18: 0)	EDA, PPG, ST	TSST	Anxiety/no anxiety	STAI	NN	N/R	96
Chen et al. (2021) [28]	34 (17: 17)	EEG	Task-rest cycle	HC/anxiety	N/R	SVM: RB F + OVO	N/R	92
Gonzalez- Carabarin et al. (2021) [23]	24 (24: 0)	EEG, ECG	Stress- inducing protocol	Mild/moderate/severe	N/R	K-means for EEG + SVM KNN DT RF	N/R	79,787,169
Ihmig et al. (2020) [15]	57 (0: 57)	ECG, EDA, RSP6 features	Exposure therapy	Low/high low/medium/high	SB	Bagged trees	10-fold cross validation	8974
Perpetuini et al. (2020) [26]	102 (102: 0)	PPG 4 features (including the gender)	N/R	N/R	STAI	GLM	Leave-one-out cross validation	
Rodríguez-Arce et al. (2020) [27]	21 (21: 0)	ST, EDA, oximetry, RSP, HR6 features	Stress- inducing protocol	Anxiety/no anxiety	STAI	SVM KNNLogR RF	10-fold cross validation	98,959,588
Xie et al. (2020) [32]	20 (10: 10)	EEG	Task-restcycle	HC/anxiety	N/R	BN + CNN2BN + DBNBN + LDAPL + LDA	N/R	675,563,556,267
Mozos et al. (2017) [35]	18 (18: 0)	EDA, PPG, HRV	TSST	Anxiety/no anxiety	STAI	Adaboost	N/R	79
Miranda et al. (2016) [31]	10 (10: 0)	EDA, ECG9 features	NE	Anxiety/no anxiety	Task level	SVM: RBF	Leave-one-out cross validation	Precision: 77 Recall: 38
Xu et al. (2015) [24]	39 (39: 0)	EEG, ECG, EMG, EDA15 features	Task-rest cycle	Anxiety/no anxiety	STAI	K-means+ GRNN	Leave-one-out cross validation	85

## Data Availability

Data is contained within the article.
